# Charting confidence in white matter brain charts: enabling study planning through stability validation

**DOI:** 10.1117/1.JMI.13.5.054001

**Published:** 2026-09-21

**Authors:** Michael E. Kim, Gaurav Rudravaram, Adam M. Saunders, Chenyu Gao, Karthik Ramadass, Nancy R. Newlin, Praitayini Kanakaraj, Sam Bogdanov, Angela L. Jefferson, Victoria L. Morgan, Alexandra Roche, Dario J. Englot, Susan M. Resnick, Lori L. Beason Held, Murat Bilgel, Laurie E. Cutting, Laura A. Barquero, Micah A. D’Archangel, Tin Q. Nguyen, Kathryn L. Humphreys, Yanbin Niu, Sophia Vinci-Booher, Carissa J. Cascio, Kimberly R. Pechman, Niranjana Shashikumar, The HABS-HD Study Team, Alzheimer’s Disease Neuroimaging Initiative, The BIOCARD Study Team, Zhiyuan Li, Panpan Zhang, John C. Gore, Yihao Liu, Lianrui Zuo, Yuankai Huo, Derek B. Archer, Timothy J. Hohman, L. Taylor Davis, Kurt G. Schilling, Daniel C. Moyer, Bennett A. Landman

**Affiliations:** a Vanderbilt University, Department of Computer Science, Nashville, Tennessee, United States; b Vanderbilt University, Department of Electrical and Computer Engineering, Nashville, Tennessee, United States; c Digital Informatics & Technology Solutions, Memorial Sloan Kettering Cancer Center, New York, United States; d Bioscope.ai, Boston, Massachusetts, United States; e Vanderbilt University, Medical Scientist Training Program, Nashville, Tennessee, United States; f Vanderbilt University Medical Center, Department of Neurology, Nashville, Tennessee, United States; g Vanderbilt University Medical Center, Department of Radiology and Radiological Sciences, Nashville, Tennessee, United States; h Vanderbilt University Institute of Imaging Science, Nashville, Tennessee, United States; i Vanderbilt University Medical Center, Department of Neurological Surgery, Nashville, Tennessee, United States; j National Institute on Aging, National Institutes of Health, Laboratory of Behavioral Neuroscience, Baltimore, Maryland, United States; k Peabody College of Education and Human Development, Department of Special Education, Nashville, Tennessee, United States; l Vanderbilt University, Department of Psychology and Human Development, Nashville, Tennessee, United States; m University of Kansas, Life Span Institute and Department of Psychology, Lawrence, Kansas, United States; n Vanderbilt University Medical Center, Vanderbilt Memory and Alzheimer’s Center, Nashville, Tennessee, United States; o Park University, Department of Computer Science, Parkville, Missouri, United States; p Vanderbilt University Medical Center, Department of Biostatistics, Nashville, Tennessee, United States

**Keywords:** normative modeling, magnetic resonance imaging, white matter, diffusion mRI

## Abstract

**Purpose:**

Normative modeling of quantitative brain measurements is being widely adopted as a promising method for charting population-level developmental trajectories and identifying abnormalities in groups and individuals. Generalized additive models for location, scale, and shape (GAMLSS) are a statistical framework that has been identified by the World Health Organization as a robust method for large-scale population modeling. Validating the stability of normative models is essential for appropriate benchmarking and detecting anomalies, especially for patients with neurodegenerative or developmental disorders. As these models continue to expand in the number of features and amount of data, it becomes increasingly important to assess the stability of the models and their predictions.

**Approach:**

An analytic approach is extended from previous work to assess variability of the GAMLSS framework for normative modeling of white matter measurements across the lifespan. The analytic variance and bias are compared with empirical bootstrapping estimates and those of an analogous model that is a nonparametric, data-driven analog.

**Results:**

Across all models, the analytic approach showed low model variability (<0.5% Coefficient of Variation) for lifespan trajectories that increased with age. Empirical variability of trajectories was slightly elevated by comparison, especially at the beginning of the lifespan. The trajectory variability for the data-driven analog was even larger, showing a ten-fold increase from the empirical GAMLSS variability. For individual centile prediction, the majority of data points for the analytic assessment had a mean absolute difference (MAD) in centile score prediction as z-scores that was less than 0.003 with the standard deviation of these errors below 0.06. The empirical assessment showed an elevated standard deviation (0.15) and MAD of z-score centile predictions (0.06) for the majority of datapoints. By contrast, when compared with the empirical assessment, the nonparametric, data-driven analog showed over a two-fold increase in the MAD of centile z-score predictions (0.14) and a three-fold increase in standard deviation (0.45) for the majority of data points. The divergences in variability across assessments suggest that individual centile predictions are more sensitive to random fluctuations in the data, which the nonparametric data-driven approach exacerbates.

**Conclusions:**

Results suggest that the GAMLSS brain charts are a stable method of defining and charting normative white matter development as well as planning future clinical or research studies. Overall, this study provides an assessment of GAMLSS-derived brain chart variance in a large-scale population.

## 1 Introduction

Brain charts, which were inspired by pediatric growth charts,^1^ are valuable tools that allow researchers to leverage large populations to investigate lifespan brain development and benchmark individuals or groups. Specifically, these charts are computational models that describe how quantitative measures derived from medical imaging data are expected to change across the human lifespan, providing age-specific reference distributions against which an individual’s measurements can be compared. Existing models have already allowed for identification of important developmental and aging milestones in the human brain using magnetic resonance imaging (MRI) data.^2^ These brain charts also have promising clinical applications for detecting anomalies. Group-level analyses have shown the utility of these charts for detecting quantifiable changes in structural brain measurements across a range of clinical groups.^2^^–^^4^ Furthermore, normative modeling of quantitative brain measurements has promise for detecting abnormalities at the individual level.^5^

There are several methods that are used to normatively model MRI-derived measurements, including Gaussian process regression, polynomial regression, hierarchical Bayesian regression, and others.^6^^,^^7^ One notable candidate is generalized additive models for location, scale, and shape (GAMLSS).^8^ In addition to being recognized by the World Health Organization (WHO) as a method for constructing growth curves,^9^ the GAMLSS framework provides flexibility for modeling many types of distributions. This flexibility includes the ability to model higher-order distributional moments, such as skewness and kurtosis, as well as the more standard mean and variance. Due to its utility, GAMLSS has been used widely in normative modeling for brain charts.^2^^,^^7^^,^^10^

Many previous works have focused on whole brain or gray matter measurements derived from structural MRI,^2^^,^^3^^,^^7^^,^^11^^,^^12^ with some models beginning to expand into functional MRI data.^13^^–^^15^ Brain charts for white matter (WM) derived from diffusion MRI (dMRI) data are emerging as well,^10^^,^^16^^,^^17^ which are important for charting lifespan changes in microstructural, or micron-level biological architecture, and macrostructural, or tract-level morphology, organization of white matter.^10^ Although they provide complementary information to structural MRI brain charts, WM brain charts similarly show promise in providing population references and identifying group-level abnormalities and normative trajectories. Furthermore, the study of developing microstructure allows for studying neurobiological hypotheses regarding regional myelination in the brain.^10^

**Fig. 1:**
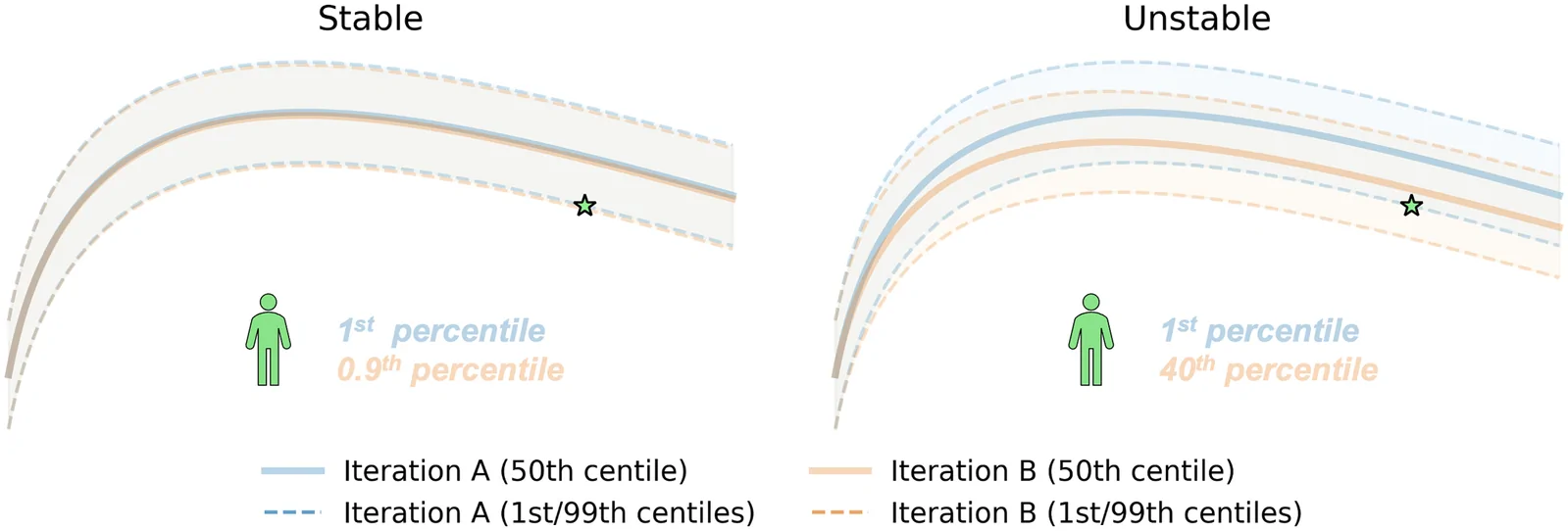
Normative models are important tools for clinical use and research to benchmark individuals against a greater population. Stability of these normative models is paramount for downstream anomaly detection, as unstable models can misclassify individuals or produce variable quantitative results during inference. (Left) In a stable normative model, confidence in individual benchmarking is high, where variability assessments yield near-identical trajectories. (Right) For unstable normative models, drastic shifts can occur due to small variations in the data, making these models much less reliable for assessing abnormalities and development.

Although these WM brain charts can provide several benefits to researchers and clinicians, validations of the stability of these models are paramount to make sure individual benchmarking and normative trajectories are robust. High variability in model fits yields low precision centile scores, reducing the utility of the brain charts (Fig. 1). In addition to model stability, there are numerous quantitative features to be extracted from dMRI, both microstructural^18^^–^^20^ and macrostructural.^21^ Coupled with the large number of potential regions of interest, collective normative WM brain charts could yield thousands of different models that require stability assessments. This high-dimensional brain phenotyping at high spatial resolution makes empirical variability assessments computationally burdensome. Furthermore, although GAMLSS has proven valuable for constructing WM brain charts, the stability and robustness of these models, especially for individual-level benchmarking, require further characterization.

To address these concerns, we conduct a characterization of the stability of these normative models for white matter created using GAMLSS. Specifically, we extend^10^ the creation of these WM brain charts for microstructure and macrostructure, to variability and stability assessments of centile scoring and normative trajectories from GAMLSS WM brain charts in two ways. First, using a standard empirical approach based on bootstrapping, and second, using an analytic approach extended from our prior work^22^ that relies on the assumptions of the GAMLSS model framework. In addition to these stability assessments, we compare the variability of our brain charts to a deep learning analog, which serves as a high-variance, nonparametric data-driven benchmark for model stability.

## 2 Methods

### 2.1 Data

We use data from our previous work^10^ consisting of N=35,120 participants with no known neurological disorders from 50 population datasets (see Table S1 in the Supplementary Material). Each participant has demographic information available for age and sex and has at least one dMRI scan; for datasets where participants received multiple scans, only one was chosen for analysis. Further details regarding data preprocessing can be found in our previous papers.^10^^,^^23^^,^^24^ Briefly, dMRI data were preprocessed for motion and distortion artifacts using the PreQual pipeline.^25^ With the MRtrix3 library,^26^ diffusion tensors were fit to the data to obtain microstructural values of fractional anisotropy (FA) and mean, radial, and axial diffusivities (MD, RD, and AxD). The TractSeg^27^ method was then used to perform subject-specific tractography for the 72 different WM tracts defined in the published algorithm. Finally, the *scilpy* Python library^28^ was used to calculate macrostructural measurements (volume, surface area, and average length) and average microstructural measurements for each tract. A manual, high-throughput quality control pipeline was used to reject failed outputs at each processing step.^23^

### 2.2 GAMLSS Normative Model Fitting

We fit 504 models in total using the GAMLSS framework,^8^ one for each combination of the 72 different WM tracts and seven microstructural/macrostructural features. The GAMLSS framework estimates the parameters μ,σ,ν,τ that define a distributional family D(μ,σ,ν,τ), where the parameters are functions of the independent variables X (age, sex, and dataset) and the dependent variable Y (WM tract measurement) is distributed according to D(μ,σ,ν,τ). This flexibility is important for capturing non-Gaussian distributions that have been shown to be present for quantitative WM metrics.^29^ For increased stability in model fitting, we normalize input age to be log-scaled, post-conception days, using the following equation:

$${\text{Age}}_{\text{normalized}}=\ln([{\text{Age}}_{\text{years}}*365.25]+280).$$ (1)

Following our previous work, D follows the generalized gamma distribution, with the probability density function GG(x;μ,σ,ν) that is implemented in the GAMLSS framework.^8^ Notably, although GAMLSS currently supports distributional families of up to four parameters, the generalized gamma distribution is parameterized by three terms. The parameters, μ, σ, and ν, roughly corresponding to the location, scale, and skewness of the distribution (see Supplementary Material for further details), are modeled as follows:

$$\log(\mu )=X\beta_{\mu }+Zb_{\mu }+U_{\mu }f_{\mu }+\epsilon_{\mu },$$ (2)

$$\log(\sigma )=X\beta_{\sigma }+Zb_{\sigma }+U_{\sigma }f_{\sigma }+\epsilon_{\sigma },$$ (3)

$$\nu =\alpha +\epsilon_{\nu },$$ (4)

where $\beta_{\left\{\mu ,\sigma \right\}}$ represents a vector for intercept and fixed effect for sex; X is the design matrix for $\beta_{\left\{\mu ,\sigma \right\}};b_{\left\{\mu ,\sigma \right\}}$ represents a random intercept vector for dataset; Z is a design matrix for $b_{\left\{\mu ,\sigma \right\}};f_{\left\{\mu ,\sigma \right\}}$ is a vector for fractional polynomial coefficients describing nonlinear age effects; $U_{\left\{\mu ,\sigma \right\}}$ is a design matrix for each $f_{\left\{\mu ,\sigma \right\}};\alpha$ is the intercept term for v; and $\epsilon_{\left\{\mu ,\sigma ,\nu \right\}}$ corresponds to the error for a given distributional parameter. Model fitting was performed using the code in the Docker for WM brain charts.^30^

### 2.3 Analytic Assessment

In GAMLSS, each block of parameters in the set θ^=[β^μβ^σb^μb^σf^μf^σα^] are estimated independently based on a backfitting process.^8^ Directly calculating V, the joint variance-covariance matrix of all fit parameters in the GAMLSS model, becomes prohibitive because each parameter batch has its own weight matrix. However, we can still estimate V as the inverse of the Hessian matrix H for the negative log likelihood l of the GAMLSS model, where

$${\left(H_{l}\right)}_{i,j}=-\frac{\partial^{2}l}{\partial \theta_{i}\partial \theta_{j}}$$ (5)

represents each entry in the Hessian matrix (see Supplementary Material section “Analytic Model – Variance-Covariance Matrix Calculation” for more details). Approximating V as $\hat{V}\approx (H_{l}{)}^{-1}$, each entry ${\hat{V}}_{i,j}$ represents the variance of the $i^{th}$ parameter when i=j and the covariance between the $i^{th}$ and the $j^{th}$ parameter when i≠j. For numerical stability, we calculate the Moore–Penrose pseudoinverse^31^ of $H_{l}$. We then model the GAMLSS parameters together as the vector $\hat{\theta }$, a k-dimensional random variable that is normally distributed, with k being the number of parameters in θ. The final, converged model estimates of each parameter $\theta_{i}$ serve as the mean of each ${\hat{\theta }}_{i}$, with $\hat{V}$ as the variance structure for the k-dimensional distribution.

Finally, to estimate the analytic variability of each model, we sample 1000 times from the k-dimensional Gaussians to obtain new models that are used in variability assessments. We choose to perform this parametric sampling procedure because Eqs. (2) and (3) employ a log-link function, meaning that the respective parameters in θ undergo a nonlinear transformation to compute the final μ and σ. This transformation will break assumptions that direct variance assessments, such as the Delta method,^32^ make regarding normality. As we have assumed θ to be normally distributed, we choose this sampling approach to fully capture the resulting nonnormal distributions of μ and σ.

### 2.4 Empirical Assessment

To obtain estimates of empirical variance for GAMLSS models, we perform bootstrap resampling for each of the 504 models. For each model, we generate 100 bootstrapped samples by sampling 95% of the data pool without replacement and refitting model parameters on each data subset. Our selection of 95% sampling follows the variability assessment of the GAMLSS models from Bethlehem et al.^2^ Each bootstrapped model is then used for downstream variability assessments.

### 2.5 Learning Analog

**Fig. 2:**
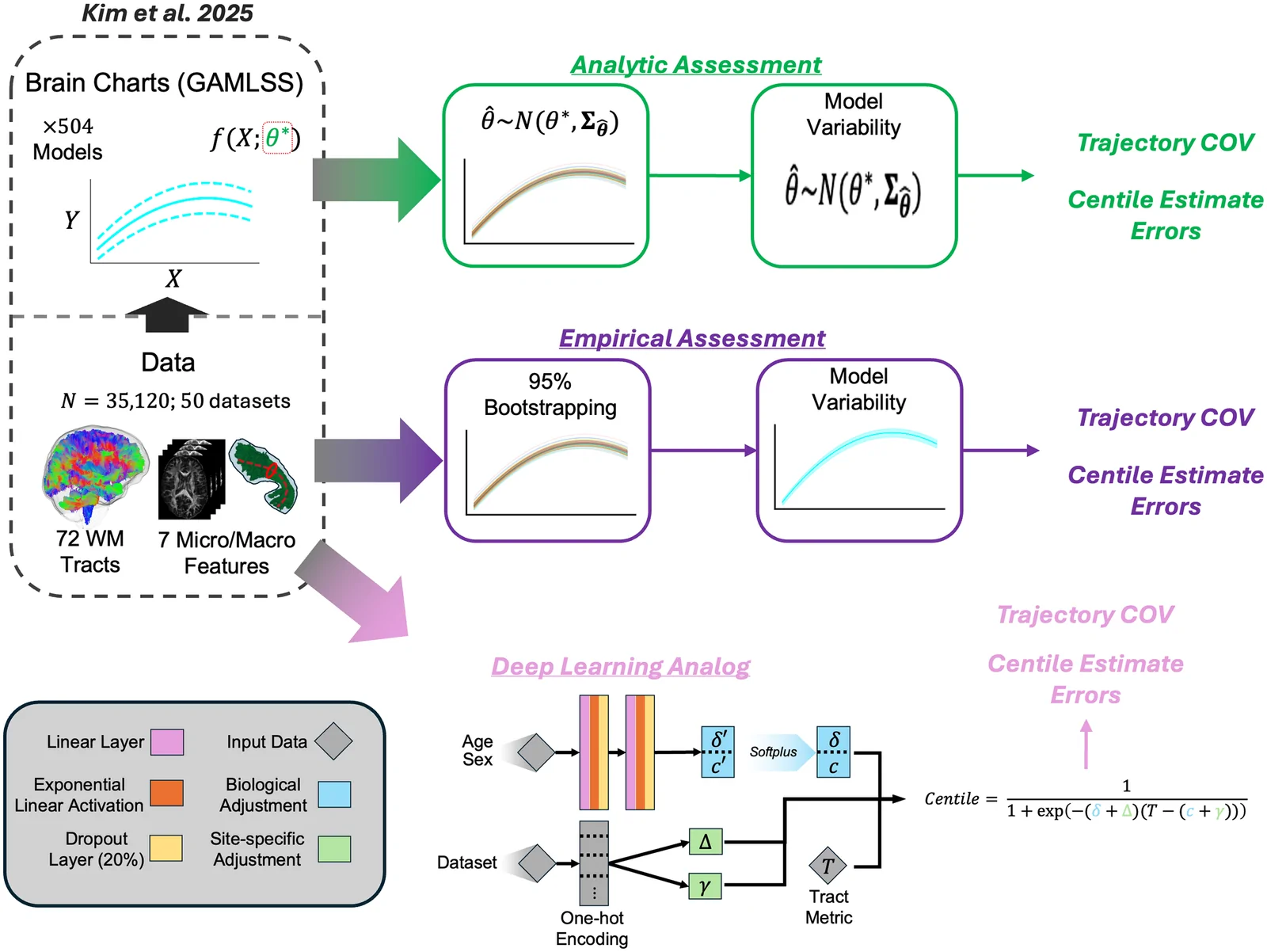
We use dMRI data from 35,120 participants to extract seven micro/macrostructural features of 72 different WM tracts that are used to create GAMLSS-based brain charts, where data pre- and post-processing have been described in our previous work. From the fit GAMLSS models (with best model fit parameters $\theta^{*}$), we conduct the analytic assessment of variability, whereas for the empirical assessment, we perform bootstrapping of models through 95% sampling without replacement. We also train deep learning analog to the GAMLSS models as nonparametric, data-driven comparisons for model variability. From each of these assessments, we assess the stability of the trajectories and the centile scoring methods by calculating the coefficient of variation (COV).

To provide a comparative benchmark for the variability observed in the GAMLSS models, we implement a deep learning architecture that performs the same normative modeling task while replacing the predefined parametric functional forms of GAMLSS with a flexible neural network. Comparing the variability of the two approaches helps place the stability of the GAMLSS models in context: If a more flexible neural network implementation of the same normative modeling task exhibits substantially greater variability when trained on the same data, this suggests that the low variability of the GAMLSS models is consistent with the regularizing effect of their statistical structure, rather than being solely a consequence of the underlying dataset. The architecture of the model is split into three parts: 1) a biological network, 2) a site network, and 3) the centile-trajectory equation (Fig. 2). For the biological network, a vector of age and sex is input to a multilayer perceptron (MLP) consisting of two hidden layers. ELU activation and 20% dropout followed each hidden layer. Age in years is normalized via a mean shift and scaling to more easily allow the network to learn age effects, whereas sex is encoded as 0 for female and 1 for male. The outputs of the biological network, δ and c, are then used for the centile trajectory equation, where δ alters the width of the learned distribution as biological covariates change and c alters the position. To make the δ and c outputs strictly positive, both are sent through a *softplus* layer.

The dataset variable is encoded as a one-hot vector that is sent as input through the site network, which is a single linear layer that outputs Δ and γ, which correspond to dataset-specific changes in δ and c, respectively. Finally, outputs of the biological and site networks, as well as the micro- or macrostructural quantitative tract metric T, are sent as inputs to the centile-trajectory equation:

$$F_{T}(t)=\frac{1}{1+e^{\left(-[(\delta +\Delta )(t-(c+\gamma ))]\right)}},$$ (6)

where t is the tract metric T normalized to the range (0,10] and $F_{T}$ is a centile value in the range (0,1). We choose $F_{T}$ to be a logistic function, as it satisfies the criteria of increasing monotonicity and a (0,1) range for a centile. Thus, the deep learning analog can be interpreted as learning a conditional logistic distribution for tract metrics, where the network predicts the distribution shape conditioned on age, sex, and site. To extract the kth centile trajectory, Eq. (6) can be rearranged as follows:

$$t=(c+\gamma )+(\frac{-1}{\left(\delta +\Delta \right)})(\ln(\frac{1}{F_{T}}-1)).$$ (7)

With this invertible architecture, the deep learning analog can be compared against the GAMLSS variability assessments for both trajectory and centile-scoring stability.

The loss function used for training is based on maximizing the likelihood of the centile-trajectory network outputs. Letting A=δ+Δ and C=c+γ, we can rewrite Eq. (6) as

$$F_{T}(t)=\frac{1}{1+e^{\left(-A(t-C)\right)}}.$$ (8)

Because Eq. (8) outputs a centile score, defined as the probability that a value is less than or equal to a given value, it corresponds to a cumulative distribution function (CDF) over the tract metric t. Taking the derivative of the CDF with respect to t yields the corresponding probability density function (PDF) $f_{T}$:

$$f_{T}(t)=\frac{A*e^{\left(-A(t-C)\right)}}{{\left(1+e^{\left(-A(t-C)\right)}\right)}^{2}},$$ (9)

which can be simplified using

$$1-F_{T}(t)=\frac{e^{\left(-A(t-C)\right)}}{1+e^{\left(-A(t-C)\right)}},$$ (10)

and Eq. (8) to yield

$$f_{T}(t)=A*(F_{T})(1-F_{T}).$$ (11)

Models are optimized by minimizing the negative of Eq. (11). Each deep learning analog is trained using the Adam optimizer with a learning rate of 1e−3 and evaluated via five-fold cross validation. During training, the data were divided into three equal batches per epoch. Training was stopped once the validation loss failed to improve for 20 consecutive epochs, and the model from that point was used for validation.

### 2.6 Assessing Model Variability

For each model of a given tract metric, we calculate the variability as the coefficient of variation (COV) (Fig. 2),

$${\mathrm{COV}}_{\text{tract}\_\text{metric}}=\frac{\sigma_{\text{tract}\_\text{metric}}}{\mu_{\text{tract}\_\text{metric}}},$$ (12)

where $\sigma_{\text{tract}\_\text{metric}}$ is the standard deviation of the model for the given tract metric we are assessing and $\mu_{\text{tract}\_\text{metric}}$ is the mean. There are two ways in which we assess variability of the brain charts. First is to assess the stability of the normative population model trajectories, which describe the ways in which the quantitative white matter measurements change throughout the lifespan. Second is to assess the stability of the centile scoring of the normative models, as stability in the centile scoring process is essential for proper anomaly detection.

We calculate the COV at different ages across the lifespan for the median trajectory to assess whether stability of the normative trajectories depends on the age being evaluated. For centile scoring, we calculate the mean absolute difference (MAD) of the centile and standard deviation of those errors. For the analytic assessment of variability, we evaluate the model at each of the 1000 iterations from our parametric sampling procedure and calculate the COV across these parametric samples. The empirical assessment is similar, instead using the 95% resampling bootstrapped model iterations. For the deep learning analog, we evaluate models 100 times using 5% node dropout in the biological network during evaluation.

### 2.7 Performing Power Analyses

The WM brain charts can be used to calculate power analyses for planning sample sizes of future microstructural or macrostructural analyses at any age in the lifespan, and we demonstrate how calculation of the model variability is important when estimating the necessary sample size to determine a population effect. To appropriately estimate the required number of participants, it is important to account for the model variability in centile scoring:

$$\sigma_{\text{total}}=\sqrt{\sigma_{\text{model}}^{2}+\sigma_{\text{population}}^{2}},$$ (13)

where $\sigma_{\text{total}}$ is the total standard deviation used for a power analysis, $\sigma_{\text{model}}$ is the variability (as standard deviation) introduced by the model that comes from the stability estimates, and $\sigma_{\text{population}}$ is the population standard deviation coming from the distribution modeling of the WM brain charts (see Supplementary Material for both $\sigma_{\text{population}}$ calculation and $\sigma_{\text{total}}$ derivation). For use in a Z-test power analysis, the required sample size N to detect an effect d is given by

$$N=\frac{{\left(Z_{\alpha }+Z_{\beta }\right)}^{2}(\frac{1}{q_{0}}+\frac{1}{q_{1}})}{{\left(d/\sigma_{\text{total}}\right)}^{2}},$$ (14)

where α is the desired false positive rate, β is the desired false negative rate, $Z_{\alpha }$ is the Z-score corresponding to a two-tailed test at the α significance level, $Z_{\beta }$ is the Z-score corresponding to the desired statistical power β, and $q_{0},q_{1}$ are the proportions of the total sample size allocated to each group, with $q_{0}+q_{1}=1$.

## 3 Results

### 3.1 Stability of Model Trajectories

**Fig. 3:**
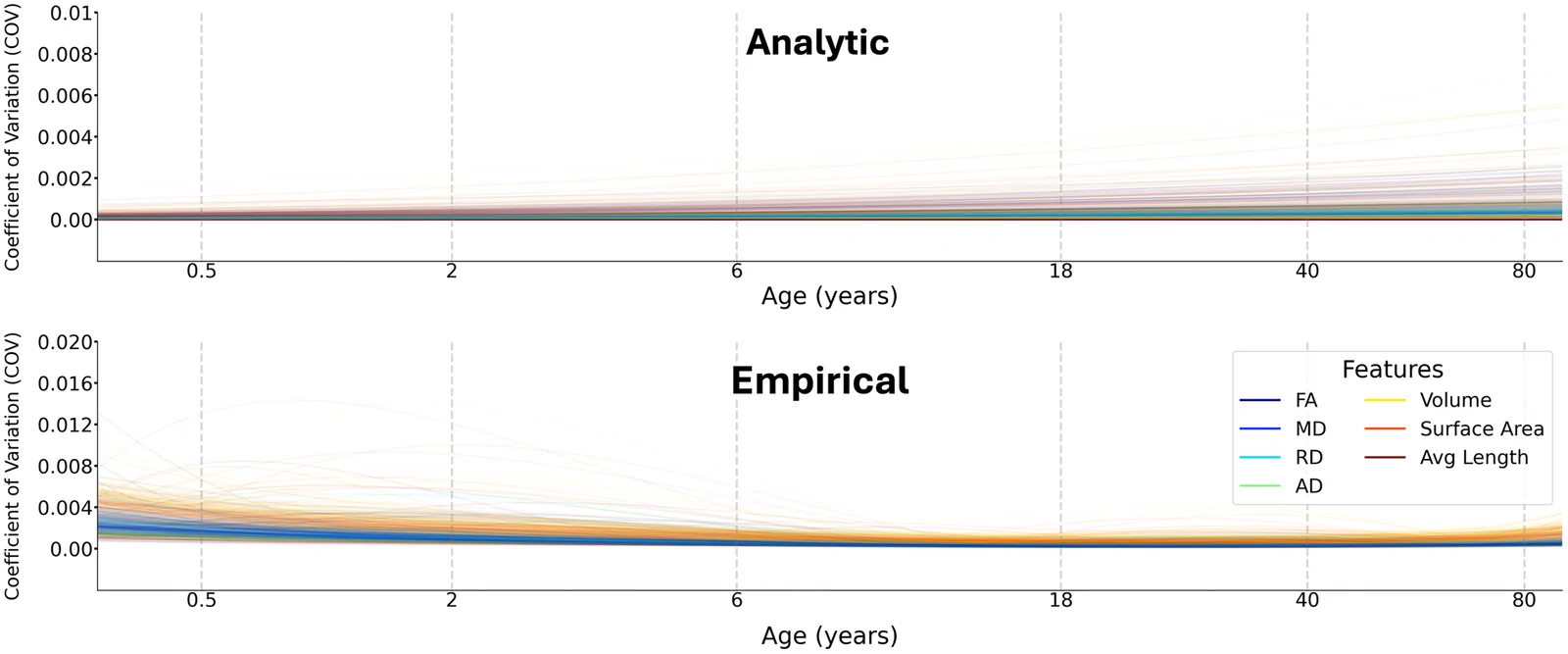
GAMLSS model trajectories are both empirically and analytically stable across the lifespan, with the COV for median trajectories sitting below 0.6% for analytic and 1.6% for empirical variability estimates. However, at most timepoints the variability of models is much lower, with the largest COV occurring at the edges of the lifespan range. This “edge effect” is a known artifact in normative modeling. We observe the edge effect to be more pronounced at older ages for the analytic analyses, whereas it is more apparent at younger ages for the empirical analysis. Shown in the plot are the COVs of all models (every tract), colored by the corresponding microstructural or macrostructural feature.

GAMLSS normative model trajectories all appear stable across the majority of the lifespan (Fig. 3). The COV for the analytic assessment is similar to the empirical assessment (<0.6%) at ages greater than 60 years but tends to yield lower variability estimates from infancy to around 40 years of age (below 0.2% for analytic and 0.8% for empirical). For the deep learning analog, while trajectories are stable across all five folds for all age ranges except 0 to 2 years (Fig. S1 in the Supplementary Material), the COV is notably increased when compared with the analytic and empirical assessments of variability. Furthermore, the COV increases more at the earliest (<10%) and oldest (<4%) ages in the lifespan when compared with the GAMLSS normative models.

### 3.2 Stability of Model Centiles

**Fig. 4:**
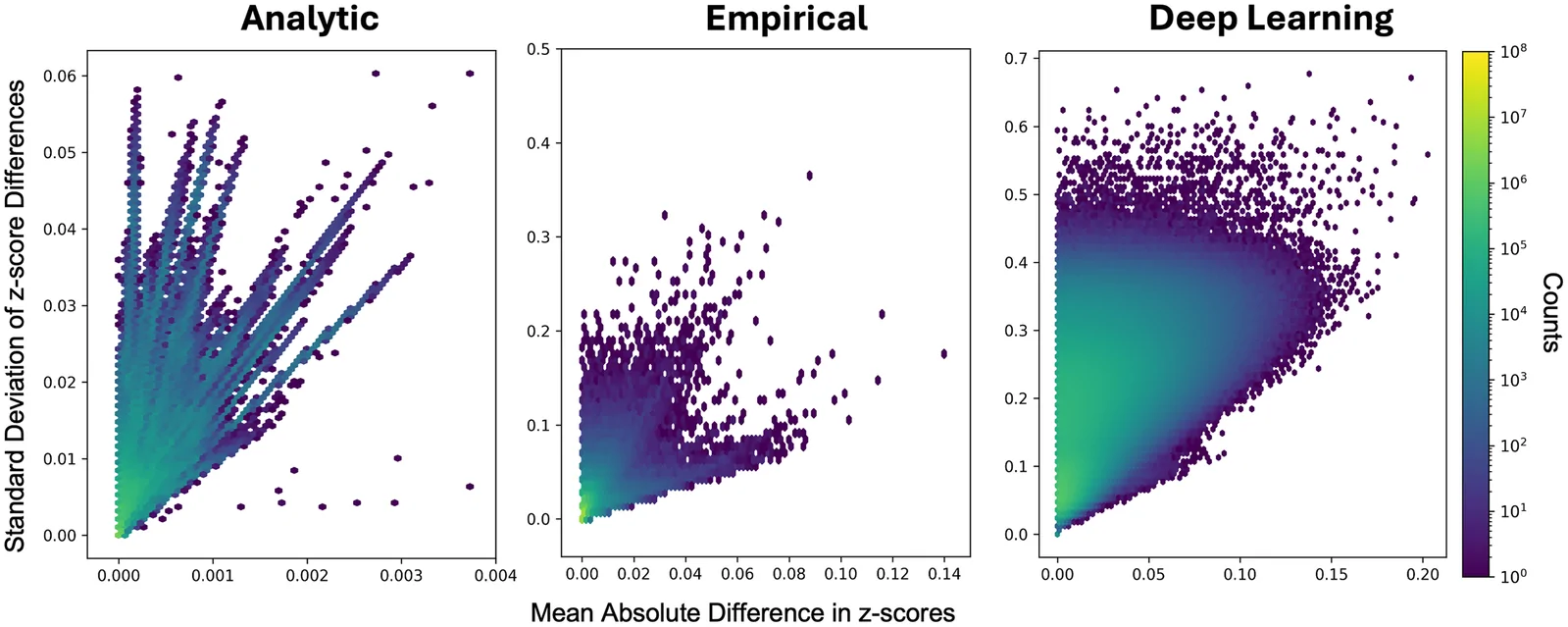
GAMLSS models demonstrate stable centile scoring across participants and data models, with mean absolute differences consistently below 0.003 and 0.08 z-scored units for analytic and empirical assessments, respectively. The variabilities in these differences are low as well, with the standard deviation for most datapoints sitting below 0.06 for analytic variability and 0.15 for empirical variability. Deep learning analogs show less stability, with mean differences mostly below 0.14 and standard deviation of differences below 0.45. However, as the deep learning analog architecture is more flexible than the GAMLSS framework, this increased variability is expected.

For centile scoring, GAMLSS models showed high stability. Centile scores from the model can be converted to Z-scores; we report the stability results for both. Mean absolute differences (MAD) in centile Z-scores were consistently below 0.003 (0.1% as centile) for the analytic assessment and 0.06 (1% as centile) for the empirical assessment (Fig. 4; Fig. S2 in the Supplementary Material). The variabilities of these differences also showed high stability, with most datapoints having a standard deviation of less than 0.06 and 0.15 for analytic and empirical, respectively, (2% as centile for both). In contrast, the deep learning analog was much less stable for centile scoring, with most datapoints having a MAD less than 0.14 (6% as centile) and standard deviation of 0.45 (15% as centile).

## 4 Discussion

We demonstrate that the recently available GAMLSS WM brain charts^30^ are highly stable models across the entire human lifespan. The notably low COV of trajectories (<1%, mostly <0.4%) for both analytic and empirical assessments indicates high confidence in the previously identified developmental and aging milestones.^10^ Furthermore, although the centile score variability for analytic and empirical assessments differed, both were highly stable in MAD and standard deviation of errors. This stability in centile scoring suggests that researchers and clinicians can be confident using the brain charts as a normative reference for benchmarking individuals. Although the edge effects we observed are common to normative modeling,^33^ the increased variability could also, in part, be due to more rapid changes in development^34^ and the beginning emergence of a variety of pathologies in older ages.^35^

As mentioned previously, analytic variability assessments offer a more rapid way to examine model stability, which is important when considering normative charts that model individual quantitative measurements for several separate regions of interest. Our results indicate that analytic variability assessments tend to yield less conservative, or smaller, estimates of model variability when compared to empirical assessments. Although prior work suggests that this divergence may be for only the most stable models,^22^ the results from this report suggest that the analytic method underpredicts variability of both normative trajectories and individual centile estimations (Figs. 3 and 4). Although we observe this dissonance between the analytic and empirical variability estimates, we note that these results are assessing only the highly stable GAMLSS WM brain charts.^7^

The increased stability of the GAMLSS brain charts compared with the deep learning analogs is expected due to the greatly increased flexibility and number of degrees of freedom that a neural network possesses, especially given the increased sensitivity that a neural network has to non-linear effects such as age. Our results suggest that the deep learning analogs, especially for individual centile prediction, are more sensitive to random fluctuations in the data due to the increased variability. In addition, the analogs were trained using 20% less data than the GAMLSS models due to the five-fold cross-validation that was used in our methodology. We also observed that the edge effects normative models suffer from Ref. 33 appeared to be exacerbated in the deep learning analogs (Fig. S1 in the Supplementary Material), which aligns with our expectations, given that the GAMLSS framework adheres to preset distributional assumptions that are not present in the analogs. These findings suggest that the modeling framework, and not solely the underlying data, contributes to the observed stability of the GAMLSS brain charts. However, as the two approaches differ in multiple respects beyond their functional parameterization, our comparison to the deep learning analogs should be interpreted as supporting evidence rather than a definitive attribution of the source of the variability. We note that we are not disparaging the use of deep learning for normative modeling; there are examples of promising works in the literature that use deep learning frameworks for normative brain modeling.^5^^,^^36^

Beyond identifying normative trajectories and performing centile scoring of new data, these WM brain charts facilitate sample size planning for future microstructural or macrostructural analyses at any age in the lifespan. Employing Eq. (13) to account for model variance, investigators can perform robust power analyses to guide study design. We provide Table 1 as an example of this, where we demonstrate power analysis results at several ages and effect sizes for the fractional anisotropy of the left arcuate fasciculus. Specifically, we use the higher empirical variability level of $\sigma_{z,\text{model}}=0.15$ as a conservative estimate for the power analysis (see Supplementary Material for translating $\sigma_{z,\text{model}}$ to $\sigma_{\text{model}}$), with a false positive rate of α=0.05, false negative rate of β=0.2, and assume that the control cohort size is equal to the experimental cohort size. We facilitate the ability to perform these power analyses by releasing the code alongside our available WM brain charts through Zenodo^10^^,^^30^ (see Supplementary Material for how to run these power analyses).

**Table 1:** Example power analysis conducted using the GAMLSS brain charts, while conservatively accounting for the model variability. The results are for the brain chart model of the mean FA of the left arcuate fasciculus (AF left).

Age	Effect Size ([FA])	Sample Size Required
8	0.020	51
8	0.010	202
8	0.005	808
15	0.020	45
15	0.010	180
15	0.005	718
25	0.020	44
25	0.010	173
25	0.005	691
50	0.020	50
50	0.010	199
50	0.005	794
65	0.020	58
65	0.010	231
65	0.005	922
80	0.020	69
80	0.010	275
80	0.005	1098

As mentioned at the beginning of this paper, normative modeling of quantitative brain measures extends beyond the cerebral white matter measures in this paper, encompassing asymmetry indices of white matter pathways,^37^ whole-brain volumetric measurements,^2^ gray matter morphology,^7^^,^^38^ and even functional and structural connectivity measures.^15^^,^^39^ These charts have been extending beyond the brain as well, with recent works encompassing morphological measures from abdominal organs such as the pancreas, liver, and spleen.^40^^,^^41^ Because these separate quantitative medical imaging normative charts have been derived from differing populations, modalities, datasets, frameworks, sample sizes, and organ systems, evaluating stability of centile scores derived from these other charts could provide valuable insight into the reliability and uncertainty of their downstream applications. More broadly, the analytic and empirical stability assessment framework presented here is not specific to white matter brain charts and could be applied to other normative modeling applications in medical imaging. Furthermore, incorporating the stability estimates of these different modality- and organ-specific models into power analyses may facilitate uncertainty-aware study planning for multimodal and multiorgan imaging studies.

There are a few considerations related to this work. Regarding the differences between the analytic and empirical variability assessments, our experiments were conducted only on the GAMLSS framework for WM normative models. Future work will need to assess if analytic methods are more appropriate for less stable models or different statistical frameworks that have been used with normative modeling, such as those detailed by Ge et al..^7^ In addition, although the GAMLSS brain charts were more stable than the deep learning variants, a more complex architecture, training method, or loss function might provide increased stability compared with the models presented in this paper, which were designed to be direct analogs to the GAMLSS brain charts. Investigating the long-term stability of this deep learning architecture, or alternative ones, represents a compelling avenue for future research.

## 5 Conclusion

We have demonstrated that previously released GAMLSS WM brain charts have high stability in both identifying normative trajectories and centile scoring of individuals using two different methods, analytic and empirical. As we found, the empirical assessment offers a more conservative estimate of model variability; researchers who wish to avoid overstating the precision of models should favor this method of assessment. Furthermore, we use a deep learning analog as a comparative baseline to highlight model stability. Our results suggest that the analytic method underestimates variability when compared to the empirical method, and GAMLSS WM brain chart stability is much greater than that of a deep learning analog. This high degree of stability suggests that sensitivity and specificity of the WM brain charts are high for individual centile predictions. Finally, when designing studies, it is important to account for age-dependent model uncertainty. As the normative WM brain charts capture this age-dependent population variability in WM microstructure and macrostructure, they have the capability to facilitate study planning. By publicly providing our method for performing power analyses with the WM brain charts while accounting for model uncertainty, we enable researchers to generate age-dependent sample size estimates unique to their own target study populations.

## Supplementary Material

10.1117/1.JMI.13.5.054001.s01

## Data Availability

Code for obtaining centile curves and fitting the GAMLSS models is available in a containerized Docker image that is downloadable at https://zenodo.org/records/15367425. Instructions for running the Docker image can also be found at that link and in the Supplementary Material. The postprocessing pipeline to obtain the microstructural and macrostructural measurements from dMRI data is also available as a Docker image downloadable at: https://hub.docker.com/r/kimm58/wm_lifespan_processing. Instructions for running the Docker image are available at https://zenodo.org/records/17144461. Code for the deep learning analog models can be found here: https://github.com/MASILab/MAGCE_WMBrainChart. Data from the Alzheimer’s Disease Neuroimaging Initiative (ADNI) are available upon request from https://adni.loni.usc.edu/. Data from the AOMIC-PIOP1 dataset are freely available for download on OpenNEURO: https://openneuro.org/datasets/ds002785/versions/2.0.0. Data from the AOMIC-PIOP2 dataset are freely available for download on OpenNEURO: https://openneuro.org/datasets/ds002790/versions/2.0.0. Data from the AOMIC-ID1000 dataset are freely available for download on OpenNEURO: https://openneuro.org/datasets/ds003097/versions/1.2.1. Data from the Boston Adolescent Neuroimaging of Depression and Anxiety (BANDA) dataset are available upon request from https://www.humanconnectome.org/study/connectomes-related-anxiety-depression. Data from BIOCARD are available upon request after filling out a data use application: https://www.gaaindata.org/partner/BIOCARD. Data from the Baltimore Longitudinal Study of Aging (BLSA) are available upon request from https://www.blsa.nih.gov/. Data from the Calgary Preschool MRI Dataset are freely available for download at: https://doi.org/10.17605/OSF.IO/AXZ5R. Data from the Centre for Attention Learning and Memory (CALM) dataset are available upon request from https://calm.mrc-cbu.cam.ac.uk/researchers/. Data from the Cambridge Center for Ageing Neuroscience (CAMCAN) dataset are available upon request from https://camcan-archive.mrc-cbu.cam.ac.uk/dataaccess/. Data from the Dallas Lifespan Brain Study (DLBS) dataset are freely available for download on OpenNEURO: https://openneuro.org/datasets/ds004856/versions/1.2.0. Data from the Health & Aging Brain Study - Health Disparities (HABS-HD) dataset are available upon request from https://apps.unthsc.edu/itr/reports. Imaging data and basic demographic information for the Healthy Brain Network (HBN) dataset are freely available to download from https://fcon_1000.projects.nitrc.org/indi/cmi_healthy_brain_network/. Phenotypic data are available upon request by filling out a data use agreement: https://fcon_1000.projects.nitrc.org/indi/cmi_healthy_brain_network/Phenotypic.html. Data from the Human Connectome Project – Aging (HCPA) dataset are available upon request from https://www.humanconnectome.org/study/hcp-lifespan-aging. Data from the Lifespan Baby Connectome Project (HCPBaby) dataset are available upon request https://www.humanconnectome.org/study/lifespan-baby-connectome-project. Data from the Human Connectome Project – Development (HCPD) dataset are available upon request from https://www.humanconnectome.org/study/hcp-lifespan-development. Data from the Human Connectome Project – Young Adult (HCP) dataset are freely available for download from https://www.humanconnectome.org/study/hcp-young-adult. Data from the Infant Brain Imaging Study (IBIS) are available for download from the National Institutes of Mental Health data archive upon request from https://nda.nih.gov/edit_collection.html?id=19. Data from the International Consortium for Brain Mapping (ICBM) dataset are available upon request from www.loni.usc.edu/ICBM. Data from the Longitudinal Brain Correlates of Multisensory Lexical Processing in Children study (shortened to Lexical in this paper) are freely available for download on OpenNEURO: https://openneuro.org/datasets/ds001894/versions/1.4.2. Data from the Memory and Aging Project (MAP), Religious Orders Study (ROS), and the Minority Aging Research Study (MARS) datasets are available upon request from https://www.radc.rush.edu/. Data from the Multisite, Multiscanner, and Multisubject Acquisitions for Studying Variability in Diffusion Weighted Magnetic Resonance Imaging (MASiVar) dataset are freely available for download on OpenNEURO: https://openneuro.org/datasets/ds003416/versions/2.0.2. Data from the National Alzheimer’s Coordinating Center (NACC) and the Standardized Centralized Alzheimer’s & Related Dementias Neuroimaging (SCAN) are available upon request from https://naccdata.org/requesting-data/data-request-process. Imaging data and basic demographic information for the Nathan Kline Institute – Rockland Sample (NKI) dataset are freely available to download from https://fcon_1000.projects.nitrc.org/indi/enhanced/sharing_neuro.html. Phenotypic data are available upon request by filling out a data use agreement: https://fcon_1000.projects.nitrc.org/indi/enhanced/sharing_phenotypic.html. Data from the Pediatric Imaging, Neurocognition, and Genetics dataset (PING) are available for download from the National Institutes of Mental Health data archive upon request from https://nda.nih.gov/edit_collection.html?id=2607. Data from the Queensland Twin Adolescent Brain (QTAB) dataset are freely available for download on OpenNEURO: https://openneuro.org/datasets/ds004146/versions/1.0.4. Data from the UCLA Consortium for Neuropsychiatric Phenomics LA5c Study (UCLA) dataset are freely available for download on OpenNEURO: https://openneuro.org/datasets/ds000030/versions/1.0.0. Data from the Southwest University (SWU) Longitudinal Imaging Multimodal dataset are freely available for download here: https://fcon_1000.projects.nitrc.org/indi/retro/southwestuni_qiu_index.html. Data from the Social Reward and Nonsocial Reward Processing Across the Adult Lifespan: An Interim Multi-echo fMRI and Diffusion Dataset (referred to as TempleSocial in this paper) are freely available for download on OpenNEURO: https://openneuro.org/datasets/ds005123/versions/1.1.3. Data from UK Biobank (UKBB) are available upon request from https://www.ukbiobank.ac.uk/. Data from the UPennRisk dataset are freely available for download on OpenNEURO: https://openneuro.org/datasets/ds002843/versions/1.0.1. Data from the dataset titled “A longitudinal neuroimaging dataset on language processing in children ages 5, 7, and 9 years old” (referred to as UTAustin579 in this paoer) are freely available for download on OpenNEURO: https://openneuro.org/datasets/ds003604/versions/1.0.7. Data from the Vanderbilt Memory and Aging Project (VMAP_JEFFERSON, VMAP_2.0, TN Aging Project) are available for download upon request from https://vmacdata.org/vmap/data-requests. Data from the Wisconsin Registry for Alzheimer’s Prevention (WRAP) are available upon request from https://wrap.wisc.edu/data-requests-2/. Data from the HEALthy Brain and Child Development (HBCD) Study are available for download upon request from (https://hbcdstudy.org/data-sharing/). Data from the Adolescent Brain Cognitive Development (ABCD) Study are available for download upon request from (https://abcdstudy.org/scientists/data-sharing/). Data used in this paper from the Ageility Project (Phase 1 only) are available for download from (https://www.nitrc.org/projects/age-ility). Data from the Early Brain Development in Twins (EBDT) dataset are available for download from the National Institutes of Mental Health data archive upon request from https://nda.nih.gov/edit_collection.html?id=2384. Data from the Bipolar & Schizophrenia Consortium for Parsing Intermediate Phenotypes dataset (BSNIP1) and its renewal (BSNIP2) are available for download from the National Institutes of Mental Health data archive upon request from https://nda.nih.gov/edit_collection.html?id=2274 and https://nda.nih.gov/edit_collection.html?id=2165. Data from the Developing Human Connectome Project (dHCP) are available for download from the National Institutes of Mental Health data archive upon request from https://nda.nih.gov/edit_collection.html?id=3955. Vanderbilt University data (MORGAN, BABIES-ABC, CUTTING, VUMC-ASD) subject to third party restrictions. Please contact corresponding authors for data requests.
