# Supplementary material for: Charting confidence in white matter brain charts: enabling study planning through stability validation
Source: J Med Imaging (Bellingham). 2026 Sep 21;13(5):054001. doi: 10.1117/1.JMI.13.5.054001 (PMC13601033; doi:10.1117/1.JMI.13.5.054001)
Supplement: Supplementary file 1 [file JMI_013_054001_SD001.pdf]

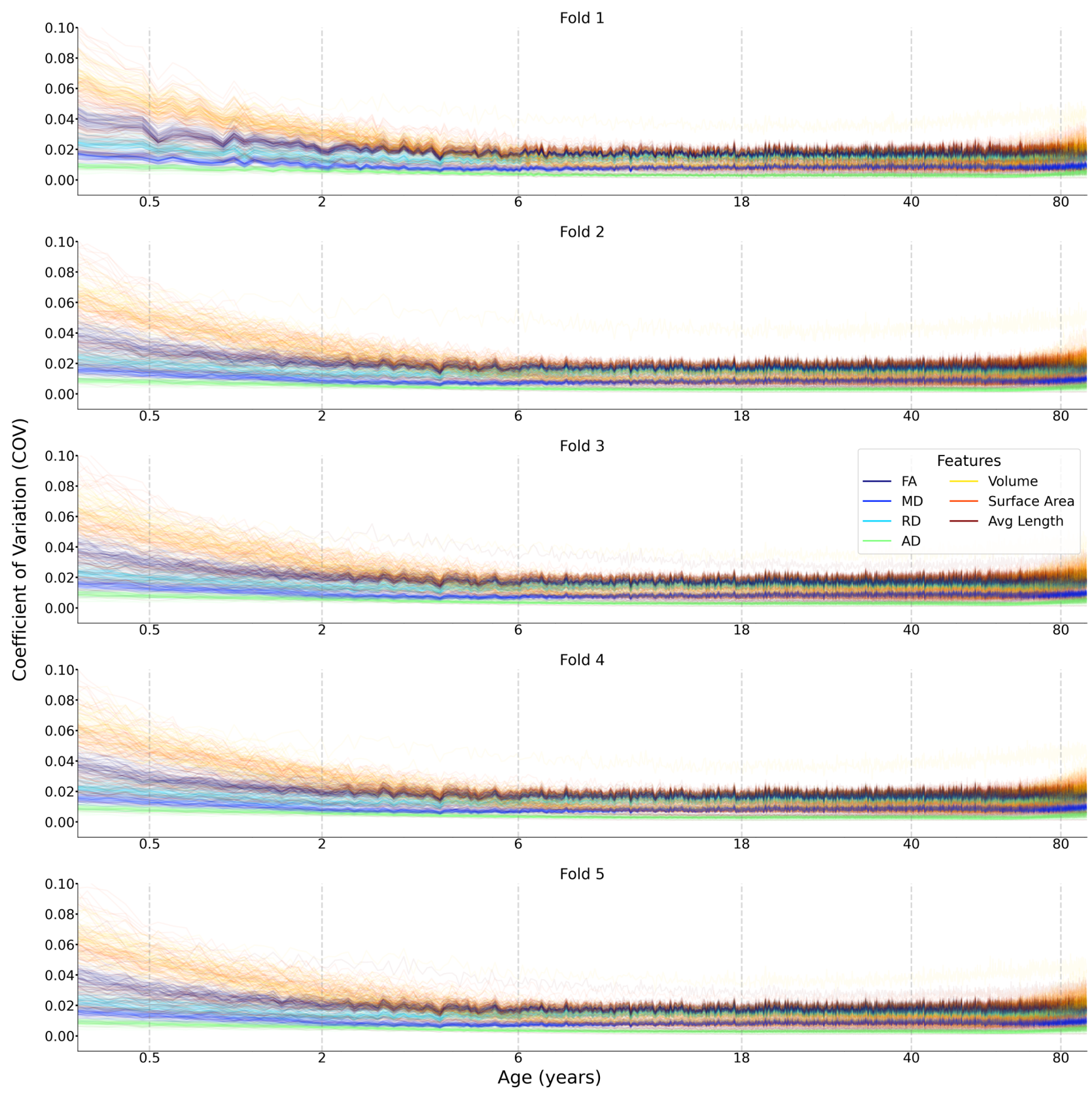

Supplemental Figure S1. Trajectories of lifespan deep learning analog models are stable across the lifespan for all folds. Edge effects are more pronounced for the deep learning analogs than the GAMLSS models, which is expected due to the larger number of degrees of freedom that the deep learning models have. We also observe macrostructural models to be less stable on average than microstructural models, which is similar to the empirical assessment of GAMLSS variability.

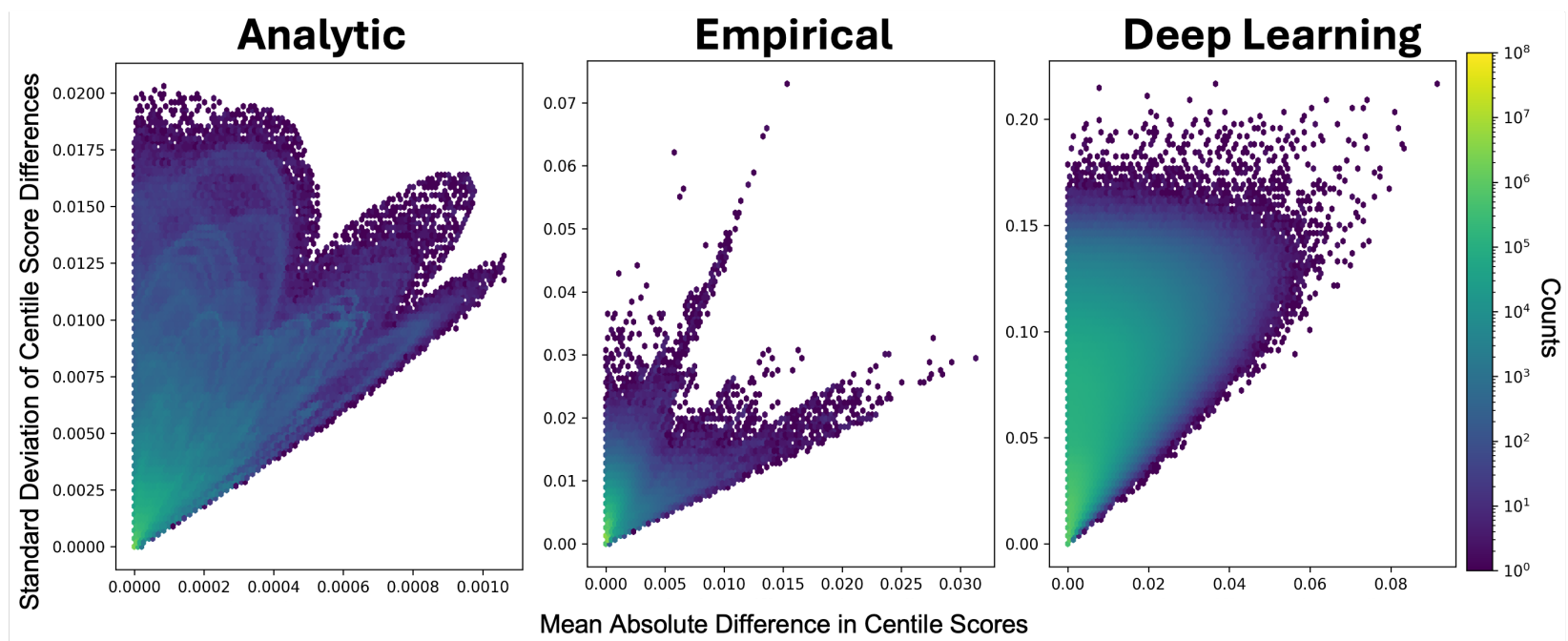

Supplemental Figure S2. GAMLSS models demonstrate stable centile scoring across participants and data models, with mean absolute differences consistently below 0.1% and 1% for analytic and empirical assessments respectively. The variabilities in these differences are low as well, with the standard deviation for most datapoints sitting below 1.75% for analytic variability and 2% for empirical variability. Deep learning analogs show less stability, with mean differences mostly below 5% and standard deviation of differences below 15%. However, as the deep learning analog architecture is more flexible than the GAMLSS framework, this increased variability is expected.

Supplemental Table S1. Summary of datasets, scans, age ranges, and participant counts. The final counts for the model fitting can be found in the "Neurotypical" column.

**Dataset**	**Age Range (years)**	**Scans**	**Participants**	**Participants After Quality Control**	**Neurotypical**
ABCD	8.3–15.3	20951	10607	10311	9000
ADNI	50.5–95.4	3451	1292	1229	584
AOMIC PIOP1	18.2–26.2	211	211	204	204
AOMIC PIOP2	18.2–25.8	226	226	224	224
AOMIC ID1000	19.0–26.0	925	925	858	858
Ageility	15.0–35.0	131	131	131	131
BANDA	14.0–16.8	207	207	207	63
BIOCARD	34.0–89.0	974	210	186	153
BLSA	22.4–94.7	5423	1035	995	942
BSNIP1	17.0–65.0	1400	594	554	197
BSNIP2	18.0–60.0	1432	730	718	233
Calgary	2.0–6.2	249	88	88	88
CALM	5.5–17.9	314	314	313	189
CAMCAN	18.5–88.9	641	641	613	613
VUMC-ASD	5.0–52.0	173	164	132	63

Continued on next page

Supplemental Table S1 – continued from previous page

Dataset	Age Range (years)	Scans	Participants	Participants After Quality Control	Neurotypical
CUTTING	5.1–16.6	1661	638	632	597
dHCP	-0.08–0.10	753	668	285	285
DLBS	21.0–89.0	955	464	444	444
EBDT	0.0–6.8	1389	468	455	455
HABSHD	50.0–90.0	3767	2762	2558	1885
HBCD	0.01–0.71	546	500	285	285
HBN	5.0–21.9	2760	2754	2519	1049
HCPA	36.0–100.0	719	719	718	718
HCPBaby	0.10–10.0	411	211	197	197
HCPD	5.6–21.9	621	621	621	621
HCP	22.0–37.0	1109	1065	1064	1064
ABC-Babies	0.04–0.59	180	142	85	85
IBIS	0.5–2.3	494	272	217	167
ICBM	19.0–80.0	264	192	185	185
Lexical	8.2–14.6	113	113	112	112
MAP	58.8–96.6	1579	589	393	273
MARS	63.1–95.5	347	184	172	143
MASIVar	5.4–8.1	118	83	83	83
MORGAN	18.0–71.0	414	256	254	110
NACC	45.1–100.1	1768	1353	677	485
NKI	6.0–85.0	2224	1289	1205	840
PING	2.0–21.7	762	762	695	695
QTAB	8.0–14.0	717	415	414	414
ROS	62.5–89.9	127	77	46	35
SCAN	42.8–93.9	303	288	260	188
SWU	17.0–27.0	460	234	231	231
TempleSocial	21.0–80.0	108	108	103	103
UCLA	21.0–50.0	262	262	248	116
UKBB	46.1–82.8	10425	10425	8509	8509
UPennRisk	18.0–34.0	251	152	152	152
UTAustin579	5.0–9.4	376	239	237	237
VMAP 2.0	50.0–90.0	342	327	266	266
VMAP	60.0–91.0	1239	328	310	160
TN Alz Project	55.0–86.0	194	168	159	58
WRAP	44.3–75.1	570	343	338	332
**Total**	**0.0–100.1**	**75036**	**46846**	**41891**	**35120**

* Ages for HBCD, ABCD-BABIES, and dHCP datasets are corrected ages based on due date.

Derivation for a Numerically Stable Generalized Gamma Variance

For the WM brain charts, a given quantitative microstructural or macrostructural variable being modeled y is assumed to follow the generalized gamma (GG)

distribution:

$$y \sim GG(\mu, \sigma, \nu) \tag{S.1}$$

where μ, σ, and ν correspond to the GG distributional parameters defined in the original GAMLSS paper [**?**]. In this parameterization, the response variable y is related to a latent variable z via:

$$y = \mu z^{1/\nu} \tag{S.2}$$

where

$$z \sim GA(1, \sigma^2\nu^2) \tag{S.3}$$

with $GA(\cdot)$ being a specific implementation of the Gamma distribution implemented in the GAMLSS framework (Rigby, 2005) [8] that has a mean of 1 and a variance of $\sigma^2\nu^2$. From this parameterization, μ roughly corresponds to the median of the distribution for y, whereas σ roughly corresponds to the dispersion and ν roughly corresponds to the skewness.
The variance of y is, by definition,

$$Var[y] = E[y^2] - (E[y])^2 \tag{S.4}$$

Plugging in our equation for the representation of y, we get:

$$Var[y] = E[(\mu z^{1/\nu})^2] - (E[\mu z^{1/\nu}])^2 \tag{S.5}$$

$$Var[y] = \mu^2 E[z^{2/\nu}] - (\mu E[z^{1/\nu}])^2 \tag{S.6}$$

Unfortunately, we could not find documentation on the implementation of the $GA(\cdot)$ distribution to calculate an arbitrary p^{th} moment from μ and σ. Instead, we can convert the GAMLSS implementation to a more traditional form of the Gamma distribution, defined as:

$$X \sim Gamma(k, \theta) \tag{S.7}$$

where the variable X has a mean of $k\theta$ and variance of $k\theta^2$, with k as a shape parameter and θ as a scale parameter. By setting corresponding equations for mean and variance equal to each other, we can solve for k and θ in terms of ν and σ:

$$\text{(mean)} \quad 1 = k\theta \tag{S.8}$$

$$\theta = \frac{1}{k} \tag{S.9}$$

$$\text{(variance)} \quad \nu^2\sigma^2 = k\theta^2 \tag{S.10}$$

$$\nu^2\sigma^2 = k\left(\frac{1}{k}\right)^2 \tag{S.11}$$

$$k = \frac{1}{\nu^2\sigma^2} \tag{S.12}$$

$$\theta = \nu^2\sigma^2 \tag{S.13}$$

We can then use the equation for the pth moment of a random variable X following the $Gamma(k, \theta)$ distribution, defined as:

$$E[X^p] = \theta^p \frac{\Gamma(k+p)}{\Gamma(k)} \tag{S.14}$$

where $\Gamma(x) = (x-1)!$ is the gamma function. Plugging in the values of k and θ in terms of σ and ν for $E[z^{1/\nu}]$, we have:

$$E[z^{1/\nu}] = \left(\frac{1}{k}\right)^{1/\nu} \frac{\Gamma\left(k+\frac{1}{\nu}\right)}{\Gamma\left(k\right)} \tag{S.15}$$

For numerical stability in our implementation, we instead calculate the log value of this p^{th} moment, leading to:

$$\log(\mu E[z^{1/\nu}]) = \log\left[\mu \left(\frac{1}{k}\right)^{1/\nu} \frac{\Gamma\left(k+\frac{1}{\nu}\right)}{\Gamma\left(k\right)}\right] \tag{S.16}$$

$$\log(\mu E[z^{1/\nu}]) = \log\left[\mu k^{(-1/\nu)} \frac{\Gamma\left(k+\frac{1}{\nu}\right)}{\Gamma\left(k\right)}\right] \tag{S.17}$$

$$\log(\mu E[z^{1/\nu}]) = \log(\mu) - \frac{1}{\nu}\log(k) + \text{lgamma}\left(k+\frac{1}{\nu}\right) - \text{lgamma}(k) \tag{S.18}$$

where $\text{lgamma}(\cdot)$ is the log of $\Gamma(\cdot)$. By a similar derivation for $\mu^2 E[z^{2/\nu}]$, we get:

$$\log(\mu^2 E[z^{2/\nu}]) = 2\log(\mu) - \frac{2}{\nu}\log(k) + \text{lgamma}\left(k+\frac{2}{\nu}\right) - \text{lgamma}(k) \tag{S.19}$$

Note that we can exponentiate both Eqs. S.18 and S.19 to obtain the original $\mu E[z^{1/\nu}]$ and $\mu^2 E[z^{2/\nu}]$ respectively. Putting the exponentiated Eqs. S.18 and S.19 together in Eq. S.4, we can then calculate the variance of the GG distribution, $Var[y]$ in a numerically stable way:

$$Var[y] = \exp\left(\log\left(E[y^2]\right)\right) - \exp\left(\left(\log((E[y])^2\right)\right) \tag{S.20}$$

Power Analysis Using White Matter Brain Charts Considering Model Stability

As mentioned in **Figure 4**, empirical methods for estimating variability of the WM brain charts captured most data having a standard deviation of less than 0.15 for z-scored centile estimates of individuals across all models. Thus, as a conservative estimate, we express the additional variability introduced by the

model (in the z-scored domain) as this standard deviation, or $\sigma_{z,model} = 0.15$. To perform a more interpretable power analysis, we can transform this value back into the original units of the quantitative variable in question.

For example, we can take the age- and sex-stratified WM brain charts to calculate the population standard deviation at a given point in the lifespan, $\sigma_{population}$, using Equation S.20. Then, by definition of a z-score, we can transform $\sigma_{z,model}$ to be in the units of the brain chart of interest via:

$$\sigma_{model} = \sigma_{z,model} * \sigma_{population} \tag{S.21}$$

Finally, we can use Equation 12 from the main manuscript to calculate the adjusted population standard deviation, σ_{total}, which can be used in a power analysis.

We provide a python script, `model_zpower_analysis.py`, to perform these power analyses that is added to our existing release of the WM brain charts at `10.5281/zenodo.15367425`. Briefly, the power analysis script can be run as follows (after downloading and setting up the Docker image, following the instructions at the Zenodo page):

```
docker run --rm \
-v </Path/to/output/directory>:/OUTPUTS \
-v </Path/to/model_zpower_analysis.py>:/model_zpower_analysis.py \
r_lifespan_env \
python3 /model_zpower_analysis.py \
<tract> <measure> --ages <ages> \
--effect_sizes <effect_sizes> \
--out_csv /OUTPUTS/power_analysis.csv
```

where `</Path/to/output/directory>/power_analysis.csv` will be the output table that is similar to **Table 1** in the main manuscript. As for the specific arguments (denoted by `<...>`):

- `</Path/to/output/directory>`: path to a directory that you wish to save the output table in
- `</Path/to/model_zpower_analysis.py>`: path to the power analysis python script on your local filesystem
- `<tract>`: name of the TractSeg tract of interest, coming from the selection defined in the TractSeg github page here: `https://github.com/MIC-DKFZ/TractSeg`
- `<measure>`: should be the WM measurement of interest, coming from the set {`fa-mean, md-mean, ad-mean, rd-mean, volume, surface_area, avg_length`}.
- `<ages>`: a list numbers (should range from 0 to 100) that are the ages to evaluate at for the power analysis

- `<effect_sizes>`: a list numbers that are the effect sizes to evaluate at for the power analysis, where the units should be those of the `<measure>` you have chosen

For helpful context, **Table 1** was generated using the following command:

```
docker run --rm \
-v /home/outputs:/OUTPUTS \
-v /home/model_zpower_analysis.py:/model_zpower_analysis.py \
r_lifespan_env \
python3 /model_zpower_analysis.py AF_left fa-mean \
--ages 8 15 25 50 65 80 \
--effect_sizes 0.02 0.01 0.005  \
--out_csv /OUTPUTS/power_analysis.csv
```

Furthermore, you can run the following command for additional information regarding arguments and configuration:

```
docker run --rm \
-v </Path/to/model_zpower_analysis.py>:/model_zpower_analysis.py \
r_lifespan_env \
python3 /model_zpower_analysis.py --help
```

Derivation for Model Variance in Power Analysis

Consider the centile scoring for our WM brain chart model:

$$P = T + \epsilon_{model} \tag{S.22}$$

where P is the centile score, T is the tract metric value, and ϵ_{model} is the error in the WM brain chart model. The variance in estimated centile, $Var[P]$, is then:

$$Var[P] = Var[T + \epsilon_{model}] \tag{S.23}$$

$$Var[P] = Var[T] + Var[\epsilon_{model}] + 2Cov[T, \epsilon_{model}] \tag{S.24}$$

where $Cov[T, \epsilon_{model}]$ is the covariance between T and ϵ_{model}. Assuming that the variability in the model is independent of the model variability, then $Cov[T, \epsilon_{model}] = 0$. Substituting in, we get:

$$Var[P] = Var[T] + Var[\epsilon_{model}] \tag{S.25}$$

As $Var[X] = \sigma_X^2$, we reach Eq. 13 for the total variability in model centiles.

Analytic Method - Variance-Covariance Matrix Calculation

Given the GAMLSS model in Eqs. 2, 3, and 4, we have the set of GAMLSS-estimated parameters

$$\theta = \begin{bmatrix} \beta_\mu & b_\mu & f_\mu & \beta_\sigma & b_\sigma & f_\sigma & \alpha \end{bmatrix}$$

where each component in the brackets represents a vector of parameters. Consequently, the full parameter vector θ is the concatenation of these sub-vectors, meaning the individual elements $\theta_1, \theta_2, \ldots, \theta_n$ correspond to the sequential scalar terms comprising β_μ, followed by those in b_μ, and so on. To estimate the variance-covariance matrix of our θ parameters, we calculate the observed Fisher Information matrix, or the inverse of the Hessian of the negative log-likelihood H_l, as

$$H_l = \begin{bmatrix} -\frac{d^2 l}{d\theta_1 d\theta_1} & -\frac{d^2 l}{d\theta_1 d\theta_2} & \cdots & -\frac{d^2 l}{d\theta_1 d\theta_k} \\ -\frac{d^2 l}{d\theta_2 d\theta_1} & -\frac{d^2 l}{d\theta_2 d\theta_2} & \cdots & -\frac{d^2 l}{d\theta_2 d\theta_k} \\ \cdots & \cdots & \cdots & \cdots \\ -\frac{d^2 l}{d\theta_k d\theta_1} & -\frac{d^2 l}{d\theta_k d\theta_2} & \cdots & -\frac{d^2 l}{d\theta_k d\theta_k} \end{bmatrix} \tag{S.26}$$

where k is the number of parameters in θ and the log-likelihood is

$$l = l_{GG}(y|\theta) - \frac{1}{2}\lambda_\mu b_\mu^T b_\mu - \frac{1}{2}\lambda_\sigma b_\sigma^T b_\sigma \tag{S.27}$$

with l_{GG} as the log-likelihood of the Generalized Gamma function for the training data y and parameters θ and $\lambda_\mu, \lambda_\sigma$ are the ratios of the residual variances to the random effect variances for μ and σ respectively. By calculating the inverse of H_l, we can obtain the observed Fisher Information Matrix as our proxy for the variance-covariance matrix of the θ parameters.
